# Practical Medicine

**Published:** 1881-06

**Authors:** 


### PRACTICAL MEDICINE.

### CATARRHAL DIATHESIS IN YOUNG GIRLS.

A special state of the organism has been demonstrated, constituting what we call a *catarrhal diathesis*. We observe in a certain number of young girls, even in very early youth, general impairments of health which plainly call for that term. This peculiar form of catarrh, which often came under my observation at Mont-Dore, is in reality a constitutional affection, and is, in all the extent of the term, a diathesis. Besides, it is not fully inherited, but it is connected with dispositions or tendencies which visibly depend on the constitutions of the parents.

*Etiology*.—Two facts must be considered in this respect: the health of the parents, and the hygienic surroundings of the girl. (1). Most always, the parents suffer from what we generally call a delicate constitution, a constitutional weakness, and suffer from some catarrhal affection, or die young. In many cases, they even show symptoms of adenitis, scrofulosis, or phthisis. (2). As a general rule, these young girls have been exposed to cold and dampness, either in their homes, or under exposure to the influences of a cold climate.

*Symptoms*.—These girls have often a light complexion, are pale, and their flesh is soft; they easily take cold, and they sometimes have what we call a *fatty chest* (an increased secretion of mucus in the respiratory passages). Although, excluding cases of more or less acute affections of the bronchi, auscultation reveals no important change, sometimes none at all. The digestive functions are often irregular. The general growth of the body is slow and labored, as it were. The girl is said to have a delicate constitution; her menses set in late; and, in many cases, the most important point is, an abundant leucorrhœa, very weakening, which may have started in early infancy.

As might be inferred from what precedes, the nervous element is found a good deal in the catarrhal diathesis of young girls. It is manifested by pains in various parts of the chest, by chokings, which take place without adequate causes, by the erratic and changeable character of the symptoms, by the frequency, obstinacy and the character of the cough, notwithstanding the negative results of physical examinations ; by an abundance of expectoration, alternating or existing simultaneously with a profuse leucorrhœa. To conclude, these profuse losses from the bronchi, or from the genital passages, finally ruin the constitution.

This diathesis, we are happy to say, is capable of being cured, by an intelligent observation of the laws of hygiene, by an appropriate treatment ; and age may also bring a cure.—Abstract from an Address of Dr. G. Richelot, in *L' Union Médicale*, March 3.

---

THE editor of the *Journal of Comparative Mèdicine* desires to secure as complete a list as possible of all persons practicing Veterinary Medicine in this country. No Veterinary Medical Register now exists. It would tend to unite members of the veterinary profession, and benefit them in many ways, and would be a convenience to many others, if such a register were published. All veterinarians are urgently requested to forward by postal, their names, titles and addresses. All such will receive a copy of the final list at cost rates. Address, Editor of Journal of Comparative Medicine, care W. P. Hyde & Co., 22 Union Square, New York City, N. Y.

SMALL POX AT BOULOGNE.—The small-pox statistics at Boulogne-sur-Mer during the months of January, February, and March are as follows: In the town and at the hospital there were 157 deaths, 94 of which were children. The disease has been of a virulent form, but the numbers have decreased during the last month. Over 1,600 people have been vaccinated from the calf, four having been placed at the disposal of the medical men of the town; and at a meeting of the Medical Society, April 5, it was decided to continue the vaccination, each medical man taking his turn at the work. On the morning of the 11th of April, there were only eight cases at the hospital, all going on well.

